# The effect of core stability training on the balance ability of young male basketball players

**DOI:** 10.3389/fphys.2023.1305651

**Published:** 2024-01-05

**Authors:** Jian Gong, Huiyan Gao, Jinghao Sui, Fei Qi

**Affiliations:** ^1^ Department of Physical Education, Graduate School Pukyong National University, Busan, Republic of Korea; ^2^ College of Physical Education, Henan University, Kaifeng, China; ^3^ Department of Sports Science and Physical Education, The Chinese University of Hong Kong, Hong Kong, Hong Kong SAR, China; ^4^ College of Physical Education and Health Science, Yibin University, Yibin, Sichuan Province, China

**Keywords:** core stability training, balance ability, adolescent male basketball players, traditional strength training, star excursion balance test (SEBT)

## Abstract

**Introduction:** This study aimed to investigate the impact of a 10-week Core Stability Training (CST) compared to Traditional Strength Training (TST) on the balance abilities of adolescent male basketball players.

**Methods:** Subjects (age: 15.70 ± 0.75, height: 178.4 ± 8.31, weight: 66.55 ± 8.34) were randomly assigned to either the Core Stability Training group or the Traditional Strength Training group. Three selected balance assessment indicators included the Single-leg Standing with Eyes Closed Test, Star Excursion Balance Test, and Core Four-Direction Endurance Test.

**Results:** 1) The scores were significantly different in both groups before and after the Single-leg Standing with Eyes Closed Test; (*p* < 0.01, *d* = 1.692, *d* = 1.837); 2) In the Star Excursion Balance Test, the scores of the experimental group showed significant difference (*p* < 0.05) or highly significant difference (*p* < 0.01) with an average effect size of (*d* = 1.727) when the left or right foot supported in the other directions before and after the training. However, there was no significant difference in scores in the c direction when the left foot supported (*p* > 0.05, *d* = 0.954); 3) In the Core Four-Direction Endurance Test, there were no significant differences in scores for the control group before and after training (*p* > 0.05, *d* = 0.567), while the experimental group showed significant differences in scores before and after training (*p* < 0.05, *d* = 1.889).

**Discussion:** Both CST and TST were effective in enhancing the balance abilities of adolescent basketball players. CST, in particular, demonstrated improvements in dynamic balance and agility across multiple planes. Basketball coaches are encouraged to consider incorporating CST training programs into their overall training plans for optimal balance enhancement.

## 1 Introduction

Core endurance refers to the ability of the core muscles to sustain resistance (Behm et al., 2010). Core strength training can be divided into two major components: the first involves core stability training tailored to the specific movement demands of athletes during competitions, while the other focuses on specialized core strength training for the development of core muscle strength. Primary core strength training involves learning movement patterns, emphasizing the sequence of force generation and the senses of core muscles in force generation. Its main objective is that activate core muscle groups and complete relatively simple movements, such as crunches, hip bridges, and back lifts (Balakrishnan et al., 2016). An increasing number of people recognize the significance of core functionality in sports activities. “Core stability” can generate maximum power in all types of sports, ranging from running to throwing running to throwing (Akuthota and Nadler, 2004; Kibler et al., 2006).

Good balance is almost a prerequisite for the success of any sport (Behm et al., 2008). Balance ability is a crucial factor in enhancing athletic skills, and strengthening core strength training is highly important for improving the balance of basketball players (Boccolini et al., 2013). Maintaining balance in the lower limbs during an athlete’s movement is challenging. The body’s vestibular organs are required to sense the motion, govern the contractions of core stabilizing muscles, control contractions of major muscle groups, and coordinate the sequence of force generation. Only by meeting these requirements can the kinetic chain be stable and the force be conducted in a coordinated manner, and can athletes achieve body stability in competitions (Chaiwanichsiri et al., 2005). Poor balance is a common cause of lower limb injuries in basketball players (Benis et al., 2016). Balance ability is an essential physiological function of the human body, Balance ability not merely enhances the atletic skills, moreover, it is a crucial guarantee for daily activities. (Li et al., 2000).

A 6-week core strength training plan was designed to investigate the effect of different training programs on the core strength of university athletes. Dynamic core strength training and static core strength training were respectively selected as variables, and training programs were tailored for athletes. In the 6-week comparative experiment, the final results indicated that dynamic core training was more effective than static core strength training (Bagherian et al., 2022). In terms of the combination of core strength training and specialized sports performance, basketball training was taken as an example to explicitly illustrate the influence of core strength training on athletic performance. Has explicitly illustrated the influence of core strength training on athletic performance. In basketball, which emphasizes confrontation, the movements of both offensive and defensive players are dynamic and unpredictable. Therefore, players should have a robust core muscle group to stabilize the ever-changing body postures during offensive or defensive maneuvers (Xie, 2014). Core strength plays a crucial role in controlling body balance and conducting force during human movements, signifying its importance in completing motor skills (Okada et al., 2011). Athletes’ bodies frequently switch from being stable to being unstable during games. Core strength training helps enhance athletes’ self-regulation abilities under unstable conditions and improves their balance (Kibler et al., 2006). A conclusion has been drawn from the research on soccer and basketball players, and it has shown that there is a certain relationship between core strength and dynamic balance, contributing to the prevention of sports injuries (Tsukagoshi et al., 2011).

However, many fitness coaches currently use traditional resistance training to enhance athletes’ physical capabilities. Consequently, coaches have traditionally focused on high-intensity strength training for the limbs (Y. Li, 2008). Some athletes may exhibit good strength in resistance training but perform poorly in actual competitions (Zazulak et al., 2007). In basketball, standard strength plays a dominant role in training; strength training enables athletes to improve their physical capabilities, enhance performance, and reducing the risk of injury (Boccolini et al., 2013). The balance abilities of adolescent athletes can be improved by strength training, including deep squats, leg presses, lunges, and curls. Research shows a significant improvement in the static and dynamic balance abilities of adolescent athletes through strength training (Mohammadi et al., 2012).

The distinction between core stability training and traditional resistance training has been a focal point for fitness training experts. However, there has been limited research on the effect of core stability training on the balance performance of basketball players. Therefore, this study aims to address this gap by comparing the effects of Core Stability Training (CST) and Traditional Strength Training (TST) on the improvement of balance abilities in adolescent basketball players. A scientifically designed experimental plan will be implemented to conduct a comparative analysis between core stability training and traditional strength training. The study will involve a 10-week experimental intervention, during which data will be collected and analyzed. The objective is to identify differences in the effects of core stability training and traditional strength training on the balance abilities of adolescent basketball players so as to determine the more effective method for enhancing the balance capabilities of young basketball players, and provide valuable insights for youth basketball training programs.

## 2 Materials and methods

### 2.1 Design and participants

The experimental subjects for this study consisted of 20 players from the Shen Lei Basketball Club in Shangqiu City, Henan Province. Originally, 23 athletes were planned to be recruited. But because 3 athletes were injured prior to the experiment, a total of 20 players were finally included in the study These players exhibited a positive training attitude, had frequently represented the club in competitions, demonstrated excellent performance, and dispiayed a strong dedication to training. The experimental interventions were conducted three times a week on Tuesdays, Thursdays, and Saturdays, with the intervention training taking place during the latter half of each training session to cater to the physiological development of the adolescent basketball players. To prevent too much fatigue, there was a 48-h gap between each intervention. This design aimed to ensure that players received sufficient recovery. Before the experimental intervention, a questionnaire survey was conducted among the participants. The survey results indicated that all participants possessed a keen interest in basketball and had strong training motivation. None of the participants had suffered from sports injuries over the past 2 years, nor had they used growth-promoting or stimulant-related drugs.

The age range of from participants was confined to 15 to 17, This experiment required participants to undergo static strength training. Adolescents aged 15 to 17 have relatively mature physiological and psychological development, a well-developed vascular system, and sound mental states which enable them to engage/take part in static strength training.

To ensure data reliability, Subjects from the Shen Lei Basketball Club were grouped. First, the heights of the subjects were measured and put in sequence; Secondly, the subjects were divided into the experimental group and the control group by random sampling. The experimental group received core stability training interventions (CST), while the control group underwent traditional strength training interventions (TST) (Table 1).

**TABLE 1:** Comparison of basic physical indicators between control group and experimental group.

ndicator	Control group	Experimental group	t-value	*p*-value
Age (Years)	15.60 ± 0.70	15.80 ± 0.79	−0.600	0.556
Height (cm)	175.30 ± 8.76	181.60 ± 7.83	−1.696	0.107
Weight (kg)	62.80 ± 8.39	70.30 ± 8.30	−2.009	0.060

This study adhered to the guidelines outlined in the “Helsinki Declaration” and obtained approval from the Physiology Committee of Henan University. All participants in this study provided informed consent in accordance with ethical considerations. This experiment was conducted with the consent of all participants and informed consent has been obtained by the parents and/or legal guardians of all subjects under the age of 16.

An independent samples *t*-test was conducted on the baseline data of adolescent basketball players in the control and experimental groups. The analysis revealed that there were no statistically significant differences in the baseline age and body measurement indicators between the two groups (*p* > 0.05).

### 2.2 Experimental procedure

Based on the NSCA-CSCS (National Strength and Conditioning Association Certified Strength and Conditioning Specialist) textbook, a preliminary selection of core stability training and traditional strength training programs was made (Jeffreys and Moody, 2021). As shown in Table 2.

**TABLE 2:** Experimental protocol for the experimental group.

Week	Mode	Content	Load	Rest (s)
Week 1	Isostatic and Dynamic Exercises	Four-point support with shoulder tap	15 reps * 3 sets	30
		Bent-leg four-point support with shoulder tap	15 reps * 3 sets	30
		Side plank (both sides)	30 s * 3 sets	30
		V-sit hold	30 s * 3 sets	30
Week 2	Isostatic and Static Exercises	Side plank (both sides)	30 s * 3 sets	30
		V-sit hold	30 s * 3 sets	30
	Non-isostatic Static Exercises	Suspension strap row (both sides)	15 reps * 3 sets	30
		Side suspension strap row (both sides)	15 reps * 3 sets	30
Week 3	Non-isostatic Dynamic Exercises	Yoga ball push-up	10 reps * 3 sets	30
		Yoga ball push-up with bent knees	10 reps * 3 sets	30
		Yoga ball push-up with raised hips	10 reps * 3 sets	30
		oga ball torso rotation (both sides)	10 reps * 3 sets	30
Week 4	Non-isostatic Dynamic Exercises	Suspension strap lat pull	15 reps * 3 sets	30
		Suspension strap mountain climber	15 reps * 3 sets	30
		Four-point support row (both sides)	15 reps * 3 sets	30
		Side suspension strap row (both sides)	15 reps * 3 sets	30
Week 5	Non-isostatic Dynamic Exercises	Side suspension strap hip flexion (both sides)	10 reps * 3 sets	30
		Anti-rotation horizontal push (both sides)	10 reps * 3 sets	30
		Single-arm farmer’s walk (both sides)	15 m * 3 sets	30
		Side plank with hip adduction (both sides)	5 reps * 3 sets	30

The core stability training program relies mainly on specific equipment to provide athletes with targeted core stability exercises. This allows athletes to engage in a greater number of core muscle groups to maintain trunk stability during core stability training. Core stability training requires athletes to activate more muscles, use various force-generating techniques, and respond to stimuli from multiple directions throughout the training process.

The traditional strength training programmes selected for the control group are conducted in accordance with Table 3, the chosen traditional strength training plan for the control group mainly comprises traditional bodyweight resistance exercises, alternating between upper and lower body strength training. This approach aims to enhance both upper and lower body strength while also contributing to core stability improvement (Association et al., 2021).

**TABLE 3:** Experimental plan for the control group.

Week	Training mode	Exercise	Load	Rest
1st	Bodyweight Strength Training	Push-ups	Max Reps * 10 Sets	30s
		Plank	1 min * 10 Sets	40s
2nd	Bodyweight Strength Training	Squat Jumps	20 s * 5 Sets	30s
		Push-up with Leg Extension	20 s * 5 Sets	30s
3rd	Bodyweight Strength Training	Frog Jumps	86 m * 3 Sets	1min
		Duck Walks	86 m * 3 Sets	1min
		Shuttle Run	2 Sets	1min
4th	Bodyweight Strength Training	Vertical Jump with Backboard Touch	10 reps * 6 Sets	30s
	Equipment-based Strength Training	Elastic Band Resistance Sprints	100 m * 8 Sets	40s
		Elastic Band Resistance Dribbling	Max Reps * 8 Sets	30s
5th	Bodyweight Strength Training	Sled Push	56 m * 5 Sets	40s
		Diamond Run	4 Sets	1min
	Equipment-based Strength Training	Weighted Squats	10 reps * 4 Sets	50s

The training load is controlled through heart rate. According to the heart rate zone (Draper, 2014), the maximum training heart rate for adolescents aged 15–17 is calculated to be between 203 and 205 (220—age). Given that the experimental subjects are in their adolescence, and their respiratory and cardiovascular systems are still developing, excessively high training intensity based on the maximum heart rate could potentially harm their physical development. To ensure the training quality and the safety of adolescent basketball players, the intensity of both core stability training and traditional strength training is ultimately controlled at a moderate to high intensity, specifically 70%–80% of the maximum heart rate. Participants are reminded every 30 min to place their fingers parallel to the left side of the neck artery of the sternocleidomastoid muscle, and their fingers start from the suprasternal notch. Then they are instructed to measure their pulse for 10 s, and the recorded data is multiplied by 6. If the heart rate falls within the 70%–80% of the maximum heart rate range, it indicates that the training is well-controlled. If the heart rate cannot reach or exceeds the specified range, adjustments will be made by modifying rest time to regulate heart rate.

### 2.3 Intervention measures explanation

Both groups are managed with standardized training arrangements, and the training duration is consistent. Prior to the experiment, instructions and supervision of the experimental procedures are provided by the researcher to ensure that the participants refrain from engaging in other physical training or skill learning during the experimental period. For participants who miss training sessions, alternative times are arranged for makeup training. Strict requirements are imposed on the participants’ movements during the experiment. Pre-test and post-test measurements of the experimental indicators are conducted within 1 week before the start of the experiment and within 1 week after the conclusion of the experiment, respectively. This prevents a decrease in internal validity due to an excessively long-time interval. To ensure accurate testing data, both pre-test and post-test measurements are conducted under stable physical conditions of the athletes. All training and testing are conducted indoors, and there is no need to consider weather conditions as a variable. To further minimize the probability of outliers affecting the results, balance ability tests are conducted twice before and after the experiment for both groups’ members, and the best value from the two tests is calculated, followed by *t*-test analysis using the standard deviation.

### 2.4 Evaluation methods of the experiment

#### 2.4.1 Single-leg standing with eyes closed

The participant stands naturally with hands hanging down, adjusts their breathing, and regulates their own state. When they participant feels ready to start the test, they look at the tester and nod slightly to signal their readiness. The tester then prepares for the test. Upon receiving the command from the tester, the participant closes their eyes and raises one foot slightly to aheight of 10–15 cm (usually starting with the right foot for the left foot’s test). Timing starts when the foot reaches the specified position. Throughout the test, the participant maintains the initial single-leg standing position without moving the foot’s position or leaning on any external objects. If the participant’s foot moves or if the hand touches other objects due to weight shift, the timing stops. The experiment is conducted three times for each foot, and the best result is recorded.

#### 2.4.2 Star excursion balance test (SEBT)

**FIGURE 1:**
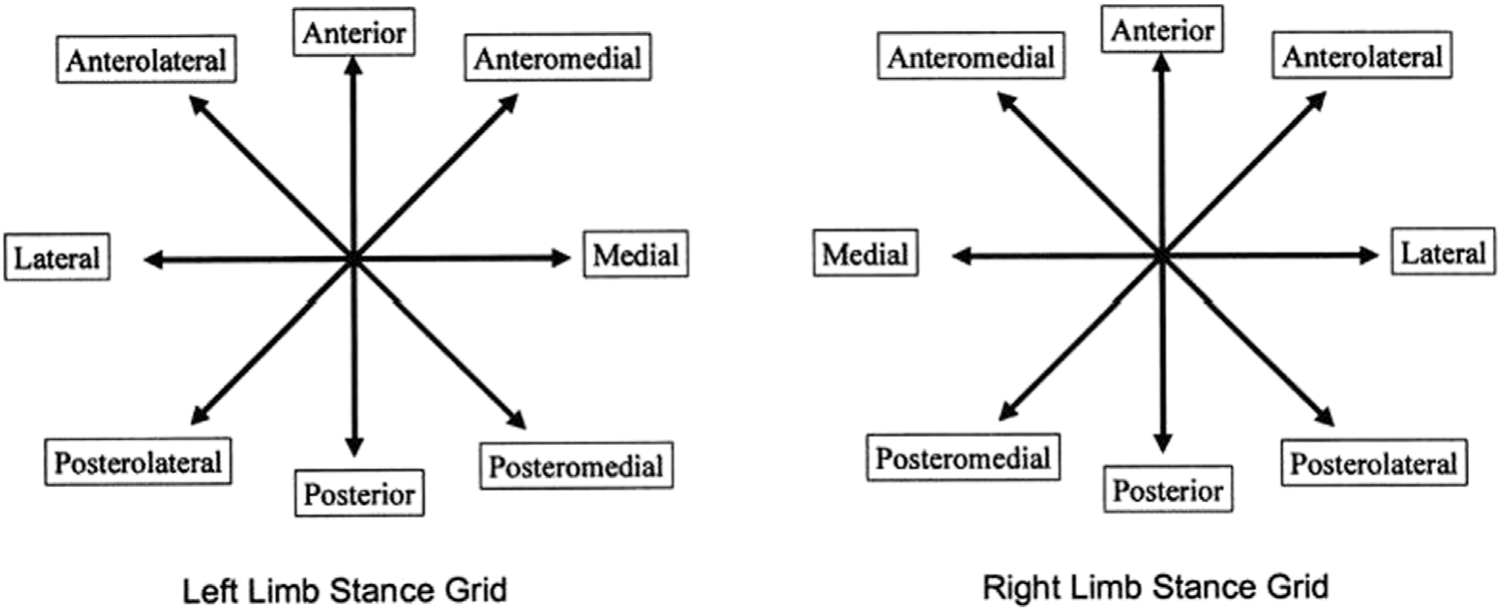
Eight-point star balance test.

The eight-point star offset balance test method is used to assess the participant’s neuromuscular control ability (Figure 1). It is widely used to test dynamic balance ability in various sports. It is easy to perform the test and the required equipment is relatively simple. The test requires the participant to maintain body stability while extending the body in eight directions. Prior to the test, the lower limb length from the lower end of the inner ankle to the lower edge of the anterior superior iliac spine is measured by a tape measure. The participant stands in the center of an eight-point star diagram facing the 12 o’clock direction. The test starts from the right leg, and then proceeds clockwise to test in 8 directions. The test leg should be fully extended in each direction, and after each direction, the test leg is returned to the center, followed by a 3-s relaxation period before testing the next direction. After testing all directions starting from the right leg, a 5-s interval is given before testing the other leg. The scale reached in each direction is recorded. Each person undergoes the test five times, and the average of the best three results is used for statistical analysis. Body balance and stability are evaluated by using the ratio of the average distance of leg extension to the entire leg length multiplied by 100. The following conditions will be considered violations: 1) the participant reports discomfort to the tester; 2) the test leg deviates from the center of the star diagram and remains off-center for more than a second after being reminded by the tester; 3) parts of the body other than the test leg touch the ground.

#### 2.4.3 Core four-direction endurance test

The subject assumes a prone position, with feet together and Anterior Superior Iliac Spine (ASIS) aligned with the edge of a bench. A tester or partner sits beneath the knees to offer support to the participant (a cushion may be employed for comfort as deemed, necessary, although precautions must be taken to prevent potential ankle discomfort). Arms are crossed anteriorly to maintain a protracted posture, with the cervical spine in a neutral alignment and the trunk parallel to the floor. Upon attaining the correct position, commencement of a countdown is initiated. Throughout the test, stringent adherence to alignment between the glenohumeral and coxofemoral joints is crucial, and the dorsum should be maintained in a slightly extended and neutral position, avoiding excessive spinal hyperextension. In the event of inadvertent spinal overextension, the tester provides a gentle verbal prompt. Persistent non-correction warrants technical intervention and subsequent recording of outcomes. Importantly, throughout the testing procedure, it is imperative to uphold the position of the shoulder joint above the level of the hip joint. Initial deviations from alignment prompt verbal reminders from the tester; recurrent deviations result in immediate cessation of timing.

### 2.5 Statistical analysis

IBM SPSS 24.0 software (IBM Corp., Armonk, NY, USA) was utilized for statistical analysis. Participants were divided into the Core Stability Training group (CST) and the Traditional Strength Training group (TST). Data from pre-tests and post-tests were collected to assess the effects of CST and TST. All variables, including the values of pre-test and post-test were measured and recorded by SPSS 24.0. We calculated the mean ± standard deviation (SD) for each variable to describe the central tendency and dispersion of the data.

To verify whether the dependent variables followed a normal distribution before and after the tests, the Shapiro-Wilk test was employed (Grover, 1977). Standard Cohen’s d was used as the effect size to measure the standardized differences between the means of the two groups. The interpretation of differences between the two groups was based on the magnitude relative to their variability, with effect sizes categorized as small (0.2–0.50), medium (0.50–0.79), large (0.80–1.29), and very large (>1.30) (Cohen, 2013). Independent t-tests were used to analyze the differences between the initial groups. Paired sample t-tests were applied to examine performance changes before and after CST and TST tests. A significance level of *p* ≤ 0.05 was set to determine the significance of the results.

## 3 Analysis of pre-test and post-test data for the three assessment indicators

### 3.1 Comparative analysis of pre-test and post-test data for dominant side single-leg stance between control and experimental groups

The paired-sample t-tests were conducted to compare the scores of Single-leg Standing with Eyes Closed before and after the intervention in both the control group (*p* = 0.003**, *d* = 1.692) and the experimental group (*p* = 0.000**, *d* = 1.837). The results revealed a highly significant difference in scores of both groups before and after the experiment (*p* < 0.01) (Table 4).

**TABLE 4:** Comparison of pre- and post-test scores for single-leg standing with eyes closed in control and experimental groups.

	Pre-test	Post-test	*p*-value	Cohen’s d
Control Group (N = 10)	17.2 ± 3.25	22.7 ± 1.94	0.003**	1.692
Experimental Group (N = 10)	14.4 ± 6.03	25.5 ± 2.91	0.000**	1.837

Note: “*” indicates significance at the 0.05 level; “**” indicates significance at the 0.01 level.

The experimental group implemented the intervention plan of CST, while the control group adopted that of TST. The core muscle group should be engaged in Single-leg Standing with Eyes Closed Test to maintain the body’s static balance. Both training protocols in the two groups incorporated exercises that require the engagement of the core muscle group to varying degrees, resulting in a certain degree of training effects on core muscle strengthening. Consequently, both groups demonstrated a highly significant improvement in the scores for Single-leg Standing with Eyes Closed Test.

### 3.2 Comparison and analysis of pre- and post-test SEBT data between control group and experimental group

The scores of the Star Excursion Balance Test (SEBT) for both the control group and the experimental group were subjected to paired sample t-tests before and after the experiment. The results for the control group showed a significant difference in the scores in the direction of H when the right foot supported (*p* < 0.05, *d* = 1.108), while there were no significant differences in scores in the other directions when both feet before and after the experiment (*p* > 0.05), with an average effect size of (*d* = 0.5902). For the experimental group, the scores showed significant difference (*p* < 0.05) or highly significant difference (*p <* 0.01) with an average effect size of (*d* = 1.727) when the left or right foot supported in the other directions before and after the experiment. However, there was no significant difference in scores in the c direction when the left foot supported. (*p* > 0.05, *d* = 0.954).

The experimental group, adopting the CST intervention plan, demonstrated significant improvements in the Star Excursion Balance Test (SEBT) scores (Table 5). A comparison between pre-intervention and post-intervention for the experimental group revealed highly significant improvements in points B, C, D, E, F, G, and H when the right foot supported. There were also significant improvements in the outer anterior and medial directions when the left foot supported. Meanwhile, highly significant improvements were seen in points D, E, F, and G when the left foot supported. After 10 weeks of CST training, the adolescent basketball players in the experimental group achieved highly significant improvements in seven directions when the right foot supported. When the left foot supported, the adolescent basketball players achieved significant improvements in two directions, and very significant improvements in four directions.

**TABLE 5:** Comparison of pre-test SEBT data between control group and experimental group.

Group	Supporting leg	Direction	Pre-test	Post-test	t-value	*p*-value	Cohen’s d
Control Group (N = 10)	Right Foot	A (Anterior)	70.4 ± 4.78	72.5 ± 5.14	−0.945	0.357	0.44
		B (Anterolateral)	60.0 ± 4.39	65.1 ± 6.62	−2.028	0.058	1.16
		C (Right)	57.5 ± 7.47	62.2 ± 9.35	−1.241	0.23	0.626
		D (Posterior)	68.0 ± 6.48	72.5 ± 7.15	−1.474	0.158	0.694
		E (Posteriomedial)	65.1 ± 9.35	70.2 ± 9.60	−1.203	0.244	0.545
		F (Posterolateral)	69.8 ± 5.43	73.8 ± 7.17	−1.405	0.177	0.736
		G (Left)	66.5 ± 5.52	71.0 ± 6.58	−1.656	0.115	0.818
		H (Anteromedial)	68.1 ± 4.58	73.2 ± 5.63	−2.221	0.039*	1.108
	Left Foot	A (Anterior)	71.3 ± 4.73	72.8 ± 4.13	−0.755	0.46	0.317
		B (Anterolateral)	69.6 ± 8.36	73.5 ± 8.73	−0.994	0.334	0.467
		C (Right)	63.0 ± 8.15	67.6 ± 9.52	−1.16	0.261	0.561
		D (Posterior)	68.6 ± 5.48	73.6 ± 6.88	−1.797	0.089	0.912
		E (Posteriomedial)	63.0 ± 7.54	66.6 ± 8.07	−1.03	0.316	0.472
		F (Posterolateral)	65.3 ± 6.68	68.2 ± 7.56	−0.908	0.376	0.433
		G (Left)	67.9 ± 5.32	71.7 ± 6.66	−1.409	0.176	0.711
		H (Anteromedial)	67.6 ± 6.76	72.3 ± 8.81	−1.377	0.198	0.687
Experimental Group (N = 10)	Right Foot	A (Anterior)	69.3 ± 5.92	72.4 ± 5.64	−1.198	0.141	0.525
		B (Anterolateral)	61.4 ± 5.44	75.3 ± 5.07	−5.906	0.001**	2.548
		C (Right)	55.8 ± 7.14	69.5 ± 9.32	−3.688	0.005**	1.918
		D (Posterior)	73.1 ± 3.44	88.8 ± 4.87	−9.57	0.006**	4.582
		E (Posteriomedial)	63.1 ± 6.64	76.9 ± 6.64	−4.647	0.001**	2.08
		F (Posterolateral)	69.2 ± 7.74	83.8 ± 8.21	−3.831	0.005**	1.879
		G (Left)	68.7 ± 5.37	83.9 ± 6.26	−6.064	0.005**	2.821
		H (Anteromedial)	68.3 ± 5.94	81.1 ± 6.53	−4.84	0.001**	2.135
	Left Foot	A (Anterior)	71.3 ± 3.49	73.8 ± 3.76	−1.538	0.246	0.718
		B (Anterolateral)	69.6 ± 7.05	78.5 ± 8.97	−2.465	0.011*	1.266
		C (Right)	61.7 ± 10.14	71.4 ± 10.88	−2.062	0.201	0.954
		D (Posterior)	66.5 ± 3.62	76.4 ± 6.48	−4.213	0.000**	2.734
		E (Posteriomedial)	62.7 ± 6.70	73.1 ± 7.76	−3.206	0.000**	1.562
		F (Posterolateral)	67.3 ± 6.49	76.5 ± 6.53	−3.156	0.001**	1.429
		G (Left)	67.5 ± 6.09	75.4 ± 7.08	−2.672	0.043*	1.294
		H (Anteromedial)	65.9 ± 4.86	74.6 ± 5.08	−3.911	0.000**	1.771

Note: “*” indicates *p* ≤ 0.05, indicating significant difference; “**” indicates *p* ≤ 0.01, indicating highly significant difference.

The control group, undergoing TST intervention for 10 weeks, exhibited improvements in all eight directions when the left or the right foot supported. However, only the scores recorded in the inner anterior direction were significantly improved when the right foot supported, while the improvements in the other 15 directions were not statistically significant.

In terms of the scores with left leg support, there was a significant difference only in the inner posterior direction between the experimental group and the control group. This suggests that the improvement in SEBT scores with the right foot support is more pronounced than that with the left foot support after 10 weeks of intervention training with different plans. The reason for this might be that athlets, who are predominantly right-handed, overly rely on the left foot support, leading to enhanced dynamic stability in the left-foot support during specialized training. Consequently, the stability in right-side support improved significantly compared to that in the left side after 10 weeks of training.

### 3.3 Comparison and analysis of core four directions before and after intervention in control and experimental groups

The scores for Core Four-Direction Endurance Test in both the control group and the experimental group were subjected to paired sample t-tests before and after the experiment. The results, as shown in Table 6, indicate that there were no significant differences in the scores for the four body positions in the control group before and after the experiment (*p* > 0.05), with an average effect size of (*d* = 0.567); However, there were significant differences in the scores for all four body positions before and after the experiment (*p* < 0.05), with an average effect size of (d = 1.889) in the experimental group. Specifically, scores for the prone position and left lateral position exhibited highly significant differences (*p* < 0.01, *d* = 2.117, *d* = 2.872).

**TABLE 6:** Comparison of abdominal muscle performance before and after intervention in control and experimental groups.

Group	Index	Pre-intervention	Post-intervention	P	Cohen’s d
Control (N = 10)	Prone Position (s)	39.4 ± 9.11	44.5 ± 11.88	0.734	0.556
	Supine Position (s)	29.1 ± 8.8	34.6 ± 10.83	0.514	0.625
	Left Lateral Position (s)	26.3 ± 6.93	30.5 ± 8.34	0.667	0.608
	Right Lateral Position (s)	32.6 ± 8.37	36.6 ± 8.95	0.593	0.479
	Prone Position (s)	39.4 ± 7.04	54.3 ± 13.35	0.000**	2.117
Experimental (N = 10)	Supine Position (s)	35.4 ± 7.11	45.9 ± 8.39	0.003**	1.472
	Left Lateral Position (s)	26.2 ± 4.42	38.9 ± 5.51	0.001**	2.872
	Right Lateral Position (s)	34.5 ± 6.95	42.1 ± 9.04	0.022*	1.094

## 4 Discussion

Among the tested indicators, Single-leg Standing with Eyes Closed and Core Four-Direction Endurance represent the body’s static balance ability. After 10 weeks of experimental intervention, TST and CST displayed different significances in the growth of scores in static balance ability tests. Ther were highly significant improvements in Single-leg Standing with Eyes test scores for both TST and CST (*p* < 0.01, *d* = 1.692, *d* = 1.837). However, the results differed in the Core Four-Direction Endurance Test. On the one hand, TST showed improvements in core stability in four abdominal directions and in four different postures after 10 weeks of traditional strength training, but the results were not statistically significant (*p* > 0.05, *d* = 0.567); On the other hand, the scores in the experimental group showed highly significant improvements in prone, supine, and left lateral positions (*p* < 0.01, *d* = 1.889), and a significant improvement in the left lateral position (*p* < 0.05, *d* = 2.872) after 10 weeks of CST. It has been observed that both Core Stability Training and Traditional Strength Training can improve the static balance ability of adolescent basketball players by analyzing the test results of Single-leg Standing with Eyes Closed and Core Four-Direction Endurance after different intervention training. However, Core Stability Training appears to be more targeted (Butcher et al., 2007; Granacher et al., 2014).

In this experiment, the SEBT, which aligns with the movement trajectory of basketball players during games, was specially employed to represent the dynamic balance ability of the body in consideration of the specific nature of basketball movements. (Fitzgerald et al., 2010). The results analysis of the SEBT provides insights into the dynamic balance abilities of adolescent basketball players undergoing CST and TST. There were significant differences in the results of the SEBT reflecting dynamic balance abilities between CST and TST. After 10 weeks of TST, there were improvements in the test scores with both right and left leg support in all eight directions, but only the scores in the internal anterior direction with the right foot support showed significant improvement (*p* < 0.05, *d* = 1.160). The improvements in scores in the other 15 directions were not statistically significant (*p* > 0.05, *d* = 0.682). After 10 weeks of CST, there were highly significant improve mentsin seven directions with the right foot support (*p* < 0.01, *d* = 2.384) and in four directions with the left foot support (*p* < 0.01, *d* = 1.874). Traditional strength training programs are exactly effective in enhancing the limb strength of adolescent basketball players, promoting the development of the neuromuscular system to some extent. However, there are limitations of traditional strength training with slow-speed muscle training in providing neural stimuli and developing vestibular organ functionality for athletes in multiple directions, angles, and movement patterns when traditional strength training is compared with Core Stability Training, But Core Stability Training, offers diverse stimuli for adolescent athletes in various aspects and movement patterns (Cressey et al., 2007; Gencer and Asma, 2017).

It is necessary to place the emphasis on thoroughly strengthening or promoting core muscles for the purpose of preventing musculoskeletal disorders, rehabilitating lumbar spine issues, and enhancing athletes’ performance (Akuthota and Nadler, 2004; Peate et al., 2007; Willardson, 2007). Activating core muscles contributes to reducing the risk of injuries, as there is always a risk of musculoskeletal injuries in young individuals engaged in sports activities. In this context, their research concludes that It is beneficial to all children by applying core stability programs to basic physical education courses (Doğan and Savaş, 2021). The skeleton of adolescent basketball players are still developing, and their muscle strength has not been fully developed yet, making them prone to injuries during both games and training. Strengthening the core stability of adolescent basketball players helps them maintain stability during sports and competitions and reduce the risk of sports injuries caused by incorrect postures and improper movements.

The CST training program for this experiment is developed according to the NSCA-CSCS textbook. It aims to create diverse training environments for athletes, and provide multi-directional and varied stimuli to the core muscle group to further enhance the athletes’ balance ability. Current research findings suggest that both types of strengthening training are equally effective interventions to improve the balance performance of adolescent basketball players. However, considering the specificity of basketball that players need to maintain control and balance while using offensive or defensive skills, it is necessary to stimulate the coordination of multiple muscle groups in the core region. In addition to the training exercises designed in this study, core stability training can also incorporate auxiliary equipment such as suspended TRX, yoga balls, balance discs, resistance bands, etc. These can be used to design training exercises that closely mimic the force generation patterns of specific technical movements making core stability training more specialized and helping athletes improve their performance.

## 5 Conclusion

A conclusion can be drawn that both CST and TST can significantly enhance the balance ability of the adolescent basketball players by analyzing the effect of CST and TST for 10 weeks on the performance of the Single-leg Standing with Eyes Closed Test among adolescent basketball players. CST demonstrates improvements in both static and dynamic balance parameters, contributing to the development of necessary balance skills. Basketball coaches can incorporate CST training programs into their training plans and implement them. Given that strength training may exert an influence on the balance abilities of basketball players, CST plans should be integrated into basketball season training as well as strength training programs.

## 6 Limitations in this study include

1) The choice of heart rate as a measure of internal load might have limitations as it did not incorporate subjective perceptions of effort as an alternative measure of internal load.
2) With regard to the statistical methods, paired-sample t-tests was employed, because there were individual differences, the sample was relatively small, and one of the aims in this study was to assess the intervention effect on the basketball players in the same group. However, it proves that variance analysis of repeated measures is more stable and it can better deal with the variance in the data. Therefore, the choice of paired-sample t-tests may affect the stability of the data.
3) The small sample size in this study restricts the generalizability of the results. Future research should consider employing larger sample sizes for validation.
4) Besides, muscle tension could potentially have an influence on the measurements of the Star Excursion Balance Test (SEBT). Therefore, muscle flexibility should be assessed before interventions.

These limitations should be taken into consideration in future research endeavors with more comprehensive and robust research designs and methodologies to enhance the reliability and generalizability of the research results.

## Data Availability

The original contributions presented in the study are included in the article/Supplementary material, further inquiries can be directed to the corresponding author.
